# Impact of aging on alanine aminotransferase levels and frailty in chronic kidney disease patients: laboratory-based cross-sectional study in Northwest Ethiopia

**DOI:** 10.3389/fmed.2025.1524459

**Published:** 2025-04-28

**Authors:** Melaku Mekonnen Agidew, Anemut Tilahun Mulu, Awgichew Behaile Teklemariam, Misganaw Asmamaw Mengstie, Addisu Melake, Edgeit Abebe Zewde, Zelalem Tilahun Muche, Samuel Berihun Dagnew, Nega Dagnew Baye, Bezawit Adane, Muluken Walle, Girma Derso

**Affiliations:** ^1^Department of Biomedical Science, College of Health Sciences, Debre Tabor University, Debre Tabor, Ethiopia; ^2^Department of Clinical Pharmacy, College of Health Sciences, Debre Tabor University, Debre Tabor, Ethiopia; ^3^Department of Anatomy, College of Medicine and Health Sciences, University of Gondar, Gondar, Ethiopia; ^4^Department of Epidemiology and Biostatistics, School of Public Health, College of Health Sciences, Injibara University, Injibara, Ethiopia; ^5^Department of Hematology and Immunohematology, School of Biomedical and Laboratory Science, College of Medicine and Health Science, University of Gondar, Gondar, Ethiopia; ^6^Amhara Regional State Health Bureau, Bahir Dar, Ethiopia

**Keywords:** chronic kidney diseases, age, alanine aminotransferase, frailty, Ethiopia

## Abstract

**Background:**

Chronic kidney disease (CKD) is a non-communicable progressive condition that leads to a gradual decline in kidney functions, resulting in different complications. Serum alanine aminotransferase (ALT) is an important biomarker for diagnosing liver comorbidities. However, ALT levels in CKD patients could be affected by aging. Despite this challenge, there is a scarcity of data on the effect of aging on frailty and ALT levels in CKD patients in Ethiopia. Thus, this study aimed to assess the impact of aging on serum levels of ALT, the magnitude of frailty, and the associated factors among CKD patients in different age groups.

**Methods:**

A hospital-based cross-sectional study involving 120 CKD patients was conducted in Bahir Dar, Ethiopia. Data were collected using structured questionnaire. Blood pressure, anthropometric parameters, ALT levels, and frailty were assessed according to standard procedures. Data were analyzed using SPSS version 25. Pearson’s correlation analysis, multiple linear analysis, and logistic regression analysis were performed to identify predictors of ALT levels and frailty, with a statistical significance level of *p* < 0.05.

**Results:**

The serum level of ALT was significantly lower in CKD patients aged ≥ 46 years compared to CKD patients aged 18–45 years. The magnitude of frailty in CKD patients aged 18–45 and ≥ 46 years was 8.5% (95% CI: 1.4–15.6) and 34.4% (95% CI: 22.5–46.4), respectively, and was significantly associated with ALT levels. Factors such as age category (AOR: 0.16, 95% CI: 0.04–0.60) and hypertension (HTN) (AOR: 10.16, 95% CI: 1.03–99.90) were significantly associated with frailty.

**Conclusion:**

The serum level of ALT was significantly correlated with age and frailty in CKD patients. Thus, ALT levels can be used as a biomarker for aging and frailty. The age of CKD patients and HTN were factors significantly associated with frailty.

## Introduction

Over 750 million people worldwide suffer from chronic kidney disease (CKD), a non-communicable disorder that leads to a gradual decline in kidney functions as characterized by various indicators. Renal dysfunction is assessed through lower levels of estimated glomerular filtration rate (eGFR), which are calculated from serum creatinine levels using the CKD Epidemiology Collaboration (CKD-EPI) equation calculator (1–3). Decreased levels of eGFR are vital markers of CKD for diagnosis and categorization (4, 5). CKD is diagnosed when one of the following criteria is met: (1) an eGFR level below 60 mL/min/1.73m^2^ for at least 3 months, irrespective of other signs of kidney damage or (2) persistent kidney damage for a minimum of 3 months, presenting with structural or functional abnormalities in kidneys with or without reduced eGFR, abnormal blood or urine composition, kidney imaging, and biopsy (6).

CKD significantly contributes to poor health outcomes and increased mortality, primarily due to its association with a range of complications, including metabolic acidosis and metabolic syndrome (7), liver disorders, sarcopenia (8), and premature aging (9, 10). Liver diseases are the most common comorbid disorders in CKD patients (8, 11) affecting many patients. Therefore, reliable liver biomarkers are needed to diagnose liver comorbidities by regular monitoring of liver functions. Increased serum levels of alanine aminotransferase (ALT) are a potential diagnostic biomarker of liver disorders (12). However, ALT levels decrease with advanced stages of CKD (13) and age (14), independent of sociodemographic and clinical factors (15). ALT activity decreases with increasing age and has the potential to serve as a novel biomarker of aging (16). It is also significantly inversely associated with frailty. Thus, the reduced levels of serum ALT in older CKD patients is an independent biomarker of aging and frailty (14).

Aging involves the decline and deterioration of cells, tissues, and organ functions, resulting in a loss of homeostasis and an inability to withstand stress-induced susceptibility to disease and mortality (17). Aging has become the most significant social and health concern worldwide (18), as it causes a gradual loss of capacity to maintain homeostasis due to structural and functional alterations that result in frailty, and it is a major risk factor for many chronic diseases (19–21). Previous studies found that aged individuals had reduced levels of ALT, although they could have a higher tendency of liver disorders (14, 16, 20), and that there is a significant correlation between aging and serum ALT activity (22). Older individuals are also more likely to experience frailty, which is an indicator of aging (20, 21).

Frailty is a condition that increases the risk of adverse health outcomes (23). It is defined based on the frailty criteria used in the Cardiovascular Health Study (CHS), which include weight loss or shrinking, weakness, exhaustion, slowness, and low physical activity (24, 25). CKD patients were considered frail if they meet at least three of the frailty criteria (24, 25). Frailty is a potential indicator of aging that has an inverse relationship with ALT levels (14, 24, 25). In aged individuals, frailty is associated with lower serum levels of ALT and acts as a potential biomarker of aging (16).

Moreover, in the Ethiopian population, there is a lack of concrete data on the effect of aging on serum levels of ALT and frailty. This gap has prompted us to explore the levels of ALT and the magnitude of frailty in CKD patients. Therefore, this study aimed to assess the impact of aging on serum levels of ALT, magnitude of frailty, and associated factors in CKD patients in Ethiopia. This could improve the early detection of liver complications in CKD patients (7, 13).

## Methods and materials

### Study area and period

This research was conducted from 1 September 2022 to 30 November 2022, in the renal clinic of Felege Hiwot Comprehensive Specialized Hospital (FHCSH) in Bahir Dar, Ethiopia. Bahir Dar is the capital city of the Amhara regional state, which is located 560 km northwest of Addis Ababa.

### Study design

We employed an institution-based cross-sectional study design to compare the levels of ALT and frailty in CKD patients admitted to the renal clinic throughout the study duration.

### Study population

This study included 120 chronically ill CKD patients who were under treatment follow-up in FHCSH renal clinic during the study period (26).

### Eligibility criteria

The current study included all CKD patients aged 18 years and above who were medically diagnosed by physicians based on their eGFR and attended the FHCSH renal clinic during the study period. However, CKD patients with a known comorbid history of any liver diseases, human immunodeficiency virus, tuberculosis, anemia, pregnant and postpartum/postnatal mothers, and patients less than 18 years old were excluded from the study.

### Sample size determination and sampling technique

#### Sample size determination

This study determined the sample size using a single population proportion formula, assuming a CKD prevalence of 12.2% (27) among medical patients with a 95% confidence interval (CI) and a 5% margin of error.


$$N\;=\;\frac{{\left(Za/2\right)}^{2}\times \left(p\right)\left(q\right)}{d^{2}}$$

where N is the minimum sample size required, Z1−*α*/2 is the standard normal variable at (1-α) % confidence level and alpha (α) level of significance. 95% CI was used, which is approximately 1.96. Based on the above-mentioned study, P is the estimated prevalence rate of CKD in the population (*p* = 0.122), and d is the margin of sampling error tolerated, which is assumed to be 0.05. Thus, the calculated sample size is 164. However, there were only 120 eligible CKD patients visiting at the renal clinic of FHCSH, and the study enrolled these 120 patients who met the eligibility criteria. These participants were divided into two groups: 18–45 years old and **≥** 46 years, to assess the difference in levels of ALT and frailty between adults and relatively older patients.

#### Sampling technique

Purposive non-probability sampling was applied to select and include all eligible CKD patients who were under treatment in the FHCSH renal clinic during the study period.

### Data collection procedure

Data were collected using a structured questionnaire that was designed by reviewing peer-reviewed published articles (4, 15, 23, 27). The questionnaire was pre-tested as a formal pilot study before the actual data collection using 5% of the study sample and evaluated by the research team to assess its soundness. The questionnaire included all the variables that can meet objectives of the study, including sociodemographic characteristics (age, sex, educational status, occupational status, and residency), behavioral features (smoking and alcohol consumption), anthropometric measurements, and clinical data (blood pressure (BP), waist circumference (WC), triacylglycerol (TAG) levels, and liver biomarkers). WC was classified as elevated as ≥ 94 cm in male patients and ≥ 80 cm in female patients, while “in normal range” was defined as < 94 cm in male patients and < 80 cm in female patients (7). TAG was also classified as “in normal range” (less than 150 mg/dL) and elevated (if it is ≥ 150 mg/dL) (7).

Anthropometric parameters were measured using weight measuring standard balance and height measuring device. Body Mass Index (BMI) was calculated from weight (kg) and height (m^2^) using the formula: BMI=Weight (kg)/Height (m^2^). World Health Organization (WHO) classified BMI as underweight (BMI <18.5 kg/m^2^), normal weight (BMI, 18.5–24.9 kg/m^2^), overweight (25.0–29.9 kg/m^2^), and obese (>30 kg/m^2^) (28). BP was directly measured using sphygmomanometer. Three BP measurements were taken with three minutes apart and the mean of the second and third readings was taken as average BP. HTN was defined as persistently elevated SBP ≥ 140 mmHg or DBP ≥ 90 mmHg, or reported uses of antihypertensive medication or known (29–32). Pre- HTN was defined as SBP between 120 and 139mmHg or DBP between 80 and 89 mmHg (32).

### Sample collection and laboratory analysis

Approximately 5 mL of blood was collected from each participant and poured into a serum separator tube (SST). This serum was separated by centrifuging the sample blood for 5 min at 5000 rpm within 40 min. Then, the serum was poured into the Nunc tube using a micropipette and stored in a refrigerator at −70°C until laboratory analysis. The laboratory analysis was carried out in accordance with the guidelines set by the expert panel from the International Federation of Clinical Chemistry (IFCC). All parameters were measured using the fully automated Roche Cobas^®^ C 502 Chemistry Analyzer (33). The normal range of AST is 0–40 IU/L for male patients and 0–32 IU/L for female patients, whereas the normal serum levels of ALT is 0–41 IU/L for male patients and 0–33 IU/L for female patients. The normal levels of direct and total bilirubin are 0.08–13.8 mg/dL and 0.146–38.0 mg/dL, respectively (33).

### Operational definition

*CKD:* Individuals are diagnosed with CKD if they meet one of the following criteria: (1) they exhibit kidney damage lasting at least 3 months, characterized by structural or functional abnormalities in kidneys, with or without decreased eGFR levels, abnormal blood or urine test results, abnormal kidney imaging findings, or biopsy results; or (2) they have consistently decreased eGFR levels (eGFR < 60 mL/min/1.73 m^2^) for a minimum of 3 months, regardless of other indicators of kidney damage (6).

*Frailty:* Frailty is defined based on the standardized criteria in clinical practice used in CHS. CKD patients were considered to have frailty if they had at least three frailty criteria (weight loss or shrinking, weakness, exhaustion, slowness, and low physical activity, as adapted for the availability of data) (24, 25).

*Aging:* Aging refers to the time-dependent decline or loss of adaptation or dysfunction that occurs with increasing age, caused by a time-progressive decline of Hamilton’s forces of natural selection. It is an insistent failure in the age-specific fitness components of individuals due to internal physiological disintegration (34, 35).

### Study variables

Liver function biomarkers and frailty were the dependent variables, while sociodemographic and behavioral factors, anthropometric parameters, and clinical factors were the independent variables.

### Data quality control

Health professional data collectors were selected based on their experience in data collection, and were well-informed on the objectives of the study, interview techniques, sample collection, and ethical considerations. The questionnaire was properly designed by researchers after reviewing different published articles (4, 15, 23, 27) related to the study objectives. It was first prepared in the English language and then translated into the local “Amharic” language by native speakers to ensure accuracy, consistency, and facilitate communication. The laboratory analysis used the standard working protocols based on the manual of the manufacturer of Chemistry Analyzer, and procedures were performed by experienced professional laboratory technologists. The collected data were revised and checked for mistakes, legibility, completeness, and consistency by the researchers before entering it into statistical analysis to clear any mistakes or ambiguity.

### Data analysis procedure

Data analysis involved checking for completeness, coding, entering into EpiData (version 3.1), and exporting to Statistical Package for Social Science (SPSS) (Version 25). Descriptive statistics were used during analysis. Variables with a *p*-value of < 0.25 in bivariable logistic regression were included in a multivariable model to identify independent associations. Adjusted odds ratios (AORs) with 95% CI were reported, with a significance level of *p* < 0.05.

## Results

### Sociodemographic characteristics of CKD patients

The mean ages of CKD patients aged 18–45 years and those aged ≥ 46 years were 33.51 ± 8.54 and 56.05 ± 5.61 years, respectively. Approximately 61.0% of the study participants aged 18–45 years and 65.6% of those aged ≥ 46 years were men. The majority of participants aged 18–45 years completed secondary education and above, whereas those aged ≥ 46 years completed primary education and below. Approximately 93.2% of the participants aged 18–45 years and 91.8% of those aged ≥ 46 years had no history of cigarette smoking, while 13.6% of the participants aged 18–45 years and 9.8% of those aged ≥ 46 years had a history of alcohol consumption (Table 1).

**Table 1:** Sociodemographic and behavioral characteristics of CKD patients aged 18–45 years and ≥ 46 years.

Variables	CKD patients aged 18–45 years and ≥ 46 years				
		CKD patients in 18–45 years (*n* = 59)		CKD patients in ≥ 46 years (*n* = 61)	
		Frequency	Percent	Frequency	Percent
Age in years (mean ± SD)		33.51 ± 8.54		56.05 ± 5.61	
Sex	Male subjects	36	61.0	40	65.6
	Female subjects	23	39.0	21	34.4
Educational status	≤ Primary education	27	45.8	49	80.3
	≥ Secondary education	32	54.2	12	19.7
Occupational status	Office workers	18	30.5	9	14.8
	Non-office workers	41	69.5	52	85.2
Residency	Rural	19	32.2	30	49.2
	Urban	40	67.8	31	50.8
Cigarette smoking	Yes	4	6.8	5	8.2
	No	55	93.2	56	91.8
Alcohol consumption	Yes	8	13.6	6	9.8
	No	51	86.4	55	90.2

Values for continuous variables are expressed in means ± SDs, and values for categorical variables are expressed in numbers and percentages. SD, Standard deviation; CKD, Chronic kidney disease.

### Anthropometric and clinical features of CKD patients

This study found that the mean values of BMI for CKD patients aged 18–45 years and≥ 46 years were 20.50 ± 2.75 and 20.82 ± 3.83 kg/m^2^, respectively. Approximately 22.0, 6.8, and 1.7% of CKD patients aged 18–45 years were underweight, overweight, or obese, respectively. In contrast, 26.2, 8.2, and 3.3% of CKD patients aged ≥ 46 years were underweight, overweight, and obese, respectively. In addition, 84.7% of CKD patients aged 18–45 years and 83.6% of those aged ≥ 46 years had elevated BP levels. Furthermore, 61.0% of CKD patients aged 18–45 years and 39.3% of those aged ≥ 46 years had end-stage renal disease (ESRD) (Table 2).

**Table 2:** Anthropometric and clinical features of CKD patients aged 18–45 and ≥ 46 years.

Variables	CKD patients in 18–45 years of age and ≥ 46 years of age				
		CKD patients in 18–45 years of age (*n* = 59)		CKD patients in ≥ 46 years of age (*n* = 61)	
		Frequency	Percent	Frequency	Percent
BMI		20.50 ± 2.75		20.82 ± 3.83	
Anthropometric features	Underweight	13	22.0	16	26.2
	Normal weight	41	69.5	38	62.3
	Overweight	4	6.8	5	8.2
	Obese	1	1.7	2	3.3
Blood Pressure	In normal range	9	15.3	10	16.4
	Elevated	50	84.7	51	83.6
WC	Increased	10	16.9	15	24.6
	In normal range	49	83.1	46	75.4
TAG	Increased	9	15.3	8	13.1
	In normal range	50	84.7	53	86.9
Group of CKD	Without ESRD	23	39.0	37	60.7
	With ESRD	36	61.0	24	39.3

Continuous variables are expressed as means ± SDs, and categorical variables are expressed as numbers and percentages. ESRD, End-stage renal disease; WC, waist circumference; TAG, triacylglycerol. CKD with ESRD includes stage 5 CKD. CKD without ESRD includes stages 1, 2, 3, and 4.

### Analysis of liver parameters of CKD patients

The overall magnitude of frailty in CKD patients was 21.7% (95% CI: 14.3–29) and the magnitude of frailty in the participants aged 18–45 years and ≥ 46 years was 8.5% (95% CI: 1.4–15.6) and 34.4% (95% CI: 22.5–46.4), respectively. The magnitude of frailty in CKD patients was statistically significantly associated with their age. After conducting normality tests, the presence of association between serum levels of liver parameters and the age of CKD patients was analyzed by using bivariate Pearson’s correlation analysis, and the age of CKD patients was significantly correlated only with the serum levels of ALT and frailty (Table 3).

**Table 3:** Serum levels of liver aminotransferases and frailty in CKD patients aged 18–45 years and ≥ 46 years.

Parameters	CKD patients aged 18–45 years and ≥ 46 years					
		CKD patients aged 18–45 years (*n* = 59)		CKD patients aged ≥ 46 years (*n* = 61)		*P*-value
		Frequency	Percent	Frequency	Percent	
Overall frailty	Yes	26 (21.7%)				
	No	94 (78.3%)				
Frailty by age	Yes	5	8.5	21	34.4	**0.0001**
	No	54	91.5	40	65.6	
AST		12.87 ± 10.81		14.32 ± 8.21		0.409
ALT		12.49 ± 9.75		8.04 ± 7.13		**0.005**
Total bilirubin		0.25 ± 0.20		0.24 ± 0.21		0.691
Direct bilirubin		0.12 ± 0.15		0.13 ± 0.17		0.823

*P-value is significant at a p-value of < 0.05. All the bold values indicate statistically significant variables.*

### Correlation analysis of liver parameters and independent variables

According to Pearson’s correlation analysis, the serum levels of AST and ALT were significantly positively correlated with eGFR and negatively correlated with creatinine and CKD stage. Similarly, ALT levels showed significant negative correlations with frailty. Total bilirubin was significantly positively associated with eGFR, whereas direct bilirubin had no significant correlation with any of the independent variables (Table 4).

**Table 4:** Correlation between liver biomarkers and independent variables in CKD patients.

Variables	Liver biomarkers							
	AST		ALT		Total bilirubin		Direct bilirubin	
	*r*	*P*-value	*r*	*P*-value	*r*	*P*-value	*r*	*P*-value
eGFR	0.864	0.000**	0.821	0.000**	0.252	0.006*	0.070	0.451
Creatinine	−0.729	0.000**	−0.607	0.000**	−0152	0.097	−0.093	0.311
Stage of CKD	−0.818	0.000**	−0.704	0.000***	−0.121	0.190	−0.073	0.426
Frailty	−0.002	0.981	−0.301	0.02*	−0.050	0.585	−0.103	0.262

*r* indicates Pearson’s correlation coefficient. *P-value is significant at ≤ 0.05. **p-value is significant at ≤ 0.001. All the bold values indicate statistically significant variables.

### Factors associated with frailty in CKD patients

This study identified age category, educational status, alcohol intake, HTN, WC, and serum TAG level as variables with a *p*-value of < 0.25 in bivariate analysis and fitted into a multiple logistic regression model to see independently significant predictor variables for frailty by using AOR with 95% CI. However, the multivariate logistic regression analysis revealed that only age category and HTN were independent significant predictors of frailty in CKD patients. CKD patients aged ≥ 46 years were more likely to experience frailty than their counterparts [AOR: 95% CI: 0.16 (0.04–0.60)]. Similarly, hypertension participants were more likely to have frailty [AOR: 95% CI: 10.16 (1.03–99-90)] (Table 5).

**Table 5:** Assessment of factors associated with frailty in CKD patients aged 18–45 and ≥ 46 years.

Variables		Presence of frailty		COR (95% CI)	AOR (95% CI)	*p*-value
		Yes	No			
Age (in years)	18–45 years*	5	54	1	1	**0.007**
	≥ 46 years**	21	40	0.18 (0.06–0.51)	0.16 (0.04–0.60)	
Educational status	≤ Primary education	22	54	1	1	0.258
	≥ Secondary education	4	40	4.07 (1.30–12.75)	2.17 (0.57–8.36)	
Alcohol intake	Yes	1	13	1	1	0.359
	No	25	81	0.25 (0.03–2.00)	0.34 (0.03–3.40)	
Presence of HTN	Yes**	8	11	1	1	**0.047**
	No*	18	83	3.35 (1.18–9.52)	10.16 (1.03–99-90)	
Waist circumference	Increased	9	16	1	1	0.712
	In normal range	17	78	2.58 (0.98–6.81)	0.64 (0.58–6-89)	
TAG level	Increased	6	11	1	1	0.882
	In normal range	20	83	2.26 (0.75–6.86)	1.19 (0.12–12.10)	

Categories having variables marked by single strikes (*) are reference categories while categories having variables marked by double strikes (**) contain significant variables to which COR and AOR were done. All the bold values indicate statistically significant variables.

## Discussion

Liver disorders are common comorbid problems in CKD patients, hence liver function biomarkers must be tested on a regular basis to detect concomitant diseases (11, 36, 37). Serum levels of AST, ALT, and bilirubin are important biomarkers for diagnosing and monitoring hepatic damage (12, 13). However, serum ALT levels tend to decrease in CKD patients aged ≥ 46 years compared to those aged 18–45 years and are not commonly elevated beyond their normal upper limit, even in the presence of liver comorbidities (14, 16). Physicians face a problem in detecting hepatic disorders in CKD patients aged ≥ 46 years as the presence of normal ALT values never confirm the absence of hepatic disease. The challenge highlights the vital need for new reference ranges for serum ALT levels in CKD patients aged ≥ 46 years to prevent hepatic diseases.

The current study found that serum levels of ALT was statistically significantly lowered when the age of CKD patients increased. Our findings are consistent with previous studies conducted in different populations (14, 16, 20). The present study also found that the mean serum level of ALT has a negative statistically significant correlation with frailty, which is one of the potential indicators of aging (20). This is in line with the findings of some previously conducted studies (14, 24, 25). However, the serum levels of AST and bilirubin were not correlated with the age of the study participants. Our findings are consistent with previous studies conducted on different populations (14, 16). The strong negative correlation between the serum level of ALT and increased age in the present study indicates that as the age of CKD patients increases, the mean value of ALT is significantly reduced. This result is in agreement with a number of previous studies conducted in different populations (14–16, 20). Hence, ALT follow a specific pattern at different age groups of CKD patients and is used as an indicator of the progression of frailty. This highlights the importance of establishing a new standard reference range for serum ALT levels to diagnose liver disorders in aged CKD patients.

The possible mechanisms for the reduction of ALT with age are not well understood. However, there are some suggested mechanisms by which ALT is reduced as the age of CKD patients increases. One of the probable causes is the reduction in some hepatic metabolic functions, which might reflect the aging of the liver and a reduction in the mass and functions of the aging liver (15, 16).

The other suggested mechanism is that there might be an anonymous lifestyle factor that confers protection to liver disease as age has a probability to affect the serum level of ALT (38). As a result, further large-scale studies are required to understand the mechanism of lowering ALT levels in CKD patients in older ages.

On the other hand, the present study showed that the overall prevalence of frailty in CKD patients was 21.7% (95% CI: 14.3–29), and the magnitude of frailty in participants aged 18–45 years and ≥ 46 years was 8.5% (95% CI: 1.4–15.6) and 34.4% (95% CI: 22.5–46.4), respectively. This higher percentage of frailty in older participants, compared to younger individuals, reveals that frailty increases with age and has a statistically significant inverse relationship with ALT levels. Our findings align with previous studies conducted on the effect of aging on frailty and ALT levels (14, 20, 24, 25). These results underscore the importance of considering age while assessing CKD patients for their liver profiles to prevent misdiagnosis.

There are various proposed factors associated with the incidence of frailty, which is considered a potential indicator of aging and is linked to the lowering of ALT levels (14, 24, 25). The research team tried to identify significant factors affecting it in the current study. Hence, the age of CKD patients was identified as a significant risk factor influencing the occurrence of frailty over their lifetime. Multiple logistic regression analysis in this study revealed that CKD patients aged ≥ 46 years were more likely to experience frailty than those aged 18–45. This finding aligns with the results of previous research that evaluated the factors affecting the presence of frailty and showed that aged individuals were more likely to be frail than younger adults (14, 20, 24, 25).

Similarly, HTN was the other factor significantly associated with frailty in CKD patients. Hypertensive CKD patients were statistically significantly more likely to have frailty problems than non-hypertensive CKD patients. The present finding aligns with the findings of other previous studies that revealed a positive correlation between frailty and hypertension (23, 39). However, our findings are inconsistent with previous studies conducted in California, which stated that the serum levels of ALT was decreased with increasing age and frailty independent of hypertension (38, 39). This discrepancy might be due to genetic and environmental influences affecting hypertension, as hypertension is hereditary (40). The genetic diversity of CKD patients in our study compared to those in California may account for the inconsistency.

## Strength and limitation of the study

The study’s key strength lies in its originality, as it is the first study assessing the correlation between ALT levels and frailty and age in CKD patients. As a result, it sheds light on the evaluation of serum levels of ALT, magnitude of frailty, and the associated factors in CKD patients in Ethiopia and serves as a foundation for future in-depth studies in the Ethiopian population to understand the mechanism by which ALT levels are reduced with age. However, the study had some limitations, such as financial constraints, that hindered sample collection and restricted the study to only 120 participants. Subsequently, this sample size and the findings may not fully represent the entire CKD population in Ethiopia to generalize the findings. In addition, the study’s cross-sectional design might prevent the observation of prospective trends, thereby impeding the establishment of causal relations between various factors examined.

## Conclusion and recommendations

Serum ALT levels were found to have a significant negative correlation with the age of CKD patients. Subsequently, it becomes a diagnostic problem and challenging to interpret ALT levels during diagnosis and management of liver disorders in older CKD patients. Hence, the research team recommends that distinct modified standard cutoff values need to be established for aged CKD patients and better to use other diagnostic methods to diagnose hepatic comorbidities in these patients. Moreover, the current study revealed that the magnitude of frailty was significantly higher in CKD patients aged ≥ 46 years compared to those CKD patients aged 18–45 years. This study also found that factors such as age of CKD patients and HTN were significantly associated with frailty.

Therefore, the present study recommends that further research with a larger sample size would be necessary to validate the results of the present study as it provides valuable preliminary data and ensures applicability to a broader population.

## Data Availability

The original contributions presented in the study are included in the article/supplementary material, further inquiries can be directed to the corresponding author.
